# Purification procedure for the isolation of a P-I metalloprotease and an acidic phospholipase A_2_ from *Bothrops atrox* snake venom

**DOI:** 10.1186/s40409-015-0027-6

**Published:** 2015-08-13

**Authors:** Danilo L. Menaldo, Anna L. Jacob-Ferreira, Carolina P. Bernardes, Adélia C. O. Cintra, Suely V. Sampaio

**Affiliations:** Departamento de Análises Clínicas, Toxicológicas e Bromatológicas, Faculdade de Ciências Farmacêuticas de Ribeirão Preto, Universidade de São Paulo, (USP), Avenida do Café, s/n, Ribeirão Preto, SP, CEP 14040-903 Brasil

**Keywords:** Snake venoms, *Bothrops atrox*, Toxins, Metalloprotease, Phospholipase A_2_, Isolation, Characterization, Chromatography

## Abstract

**Background:**

Snake venoms are complex mixtures of inorganic and organic components, mainly proteins and peptides. Standardization of methods for isolating bioactive molecules from snake venoms is extremely difficult due to the complex and highly variable composition of venoms, which can be influenced by factors such as age and geographic location of the specimen. Therefore, this study aimed to standardize a simple purification methodology for obtaining a P-I class metalloprotease (MP) and an acidic phospholipase A_2_ (PLA_2_) from *Bothrops atrox* venom, and biochemically characterize these molecules to enable future functional studies.

**Methods:**

To obtain the toxins of interest, a method has been standardized using consecutive isolation steps. The purity level of the molecules was confirmed by RP-HPLC and SDS-PAGE. The enzymes were characterized by determining their molecular masses, isoelectric points, specific functional activity and partial amino acid sequencing.

**Results:**

The metalloprotease presented molecular mass of 22.9 kDa and pI 7.4, with hemorrhagic and fibrin(ogen)olytic activities, and its partial amino acid sequence revealed high similarity with other P-I class metalloproteases. These results suggest that the isolated metalloprotease is Batroxase, a P-I metalloprotease previously described by our research group. The phospholipase A_2_ showed molecular mass of 13.7 kDa and pI 6.5, with high phospholipase activity and similarity to other acidic PLA_2_s from snake venoms. These data suggest that the acidic PLA_2_ is a novel enzyme from *B. atrox* venom, being denominated BatroxPLA_2_.

**Conclusions:**

The present study successfully standardized a simple methodology to isolate the metalloprotease Batroxase and the acidic PLA_2_ BatroxPLA_2_ from the venom of *B. atrox*, consisting mainly of classical chromatographic processes. These two enzymes will be used in future studies to evaluate their effects on the complement system and the inflammatory process, in addition to the thrombolytic potential of the metalloprotease.

## Background

Envenomation caused by snakes is a serious public health problem worldwide, especially in tropical and subtropical countries [1–4]. In 2012, according to the Brazilian Ministry of Health, around 28,000 cases of snake envenomations were reported, with the following predominance of genera responsible for accidents: *Bothrops* (72 %), *Crotalus* (7.6 %), *Lachesis* (4.5 %) and *Micrurus* (0.8 %) [5].

Snake venoms consist of inorganic compounds – including sodium, zinc, calcium and other ions – and organic components such as biogenic amines, amino acids, carbohydrates, citrates, nucleosides, as well as proteins and peptides, which correspond to more than 90 % of the dry weight of the venom. The protein components include enzymes such as phospholipases A_2_ (PLA_2_s), L-amino acid oxidases (LAAOs), serine proteases (SVSPs) and metalloproteases (SVMPs) [6, 7]. These toxins and other components of snake venoms can act independently or synergistically to cause local or systemic tissue damage and various other toxic effects [8, 9].

In order to isolate specific proteins from snake venoms, which are highly complex and may present more than 100 protein components [10], usually two or more chromatographic steps are needed, which may include steps of molecular exclusion, ion exchange, affinity, reverse phase, among others. The choice of chromatography type depends on the specific characteristics of each protein to be isolated.

The composition of snake venoms results from the interaction of several factors such as genetics, age, sex, feeding and geographic location of the specimen [11, 12]. Thus, standardization of methods for the isolation of bioactive molecules from these venoms is extremely difficult to achieve since they may vary widely in their compositions, even within the same snake species. Proteomic studies on venoms of *Bothrops atrox*, for example, showed significant variations in their protein compositions when venoms were from specimens in different stages of maturation or different geographic locations [13–15].

The snake species *B. atrox* is responsible for the majority of snakebites in the Brazilian Amazon region. In humans, envenomations by this snake cause local effects such as edema, necrosis and local hemorrhage, as well as systemic effects, including changes in blood coagulation and various bleeding sites along the bite [13]. Proteomic analyses of venoms from specimens located in Brazil have shown that metalloproteases account for more than 70 % of their protein content (~23 % of the P-I class and 49 % of the P-III class), followed by PLA_2_ with approximately 14 % (~12 % of Asp49 PLA_2_s and ~2 % of Lys49 PLA_2_s) [14].

In this context, the present study aimed to standardize a method of isolation to obtain a metalloprotease of the P-I class and an acidic phospholipase A_2_ from the crude venom of *B. atrox*, as well as to characterize and identify these molecules to enable future functional studies.

## Materials and methods

### Venom and other materials

The venom of *B. atrox*, collected from specimens found in the region of Peri Mirim, state of Maranhão, was acquired from the Center for Extraction of Animal Toxins (CETA, Morungaba, SP). Equipment and other materials used in this study are described in each specific section of the article, and reagents not otherwise specified were of analytical grade.

### Animals

Male BALB/c mice (18–22 g) were provided by the animal facilities at the University of São Paulo (USP), Ribeirão Preto, SP, Brazil, and maintained on a 12 hour-cycle at room temperature (22-25 °C) with free access to standard chow and water. Animal care procedures were performed according to the Brazilian College of Animal Experimentation (COBEA) guidelines and the experimental protocols were approved by the Committee for Ethics on Animal Use (CEUA) from FCFRP-USP (protocol number: 13.1.336.53.4).

### Isolation of toxins from *Bothrops atrox* venom

Chromatographic fractionation of *B. atrox* venom to obtain the toxins of interest began with a molecular exclusion step on Sephacryl S-200, followed by anion exchange chromatography on DEAE Sepharose. The fraction containing the metalloprotease (MP) was then ultrafiltered in a concentrator tube with membrane of MWCO 3,000, Vivaspin® 20 (Sartorius, Germany), while the fraction containing the phospholipase A_2_ (PLA_2_) was subjected to a C18 reverse phase column using ÄKTA*™* purifier system. The classical chromatography resins as well as the reverse phase column and the ÄKTA*™* system were obtained from GE Healthcare (USA).

The absorbance of the chromatographic fractions were measured at a wavelength of 280 nm, using a spectrophotometer Thermo Scientific™ GENESYS 10 UV (Thermo Fisher Scientific, Inc., USA) or the UNICORN*™* 5.11 software for the ÄKTA*™* purifier system (GE Healthcare, USA). Then, data were plotted on graphs using Origin 8 software for the obtainment and analysis of the chromatographic profiles.

#### Molecular exclusion chromatography on Sephacryl S-200

Crude and crystallized venom from *B. atrox* (350 mg) was suspended in 2 mL of 0.2 M ammonium bicarbonate buffer (AMBIC), pH 7.8, followed by centrifugation at 10,000 × *g* for ten minutes at room temperature. The clear supernatant obtained was applied to a chromatography column containing Sephacryl S-200 resin (100 × 2.6 cm), previously equilibrated and eluted with 0.2 M AMBIC buffer, pH 7.8. Fractions of 3 mL were collected per test tube, at a flow rate of 20 mL/hour at room temperature. All eluted fractions were assessed for their hemorrhagic activity and on SDS-PAGE, as described below. Chromatographic fraction S3 was selected based on its protein profile in gel and by presenting hemorrhagic activity, being lyophilized and submitted to the next chromatographic step.

#### Ion exchange chromatography on DEAE Sepharose

Fraction S3 from Sephacryl S-200 was diluted in 3 mL of 0.05 M AMBIC buffer, pH 7.8, and applied to a chromatography column containing DEAE Sepharose resin (15 × 2 cm), previously equilibrated with the same buffer. Elution of fractions was performed using three steps: 50 mL of 0.05 M AMBIC, pH 7.8; continuous concentration gradient of AMBIC from 0.05 M to 0.5 M, pH 7.8 (150 mL), and finally 100 mL of 1 M AMBIC, pH 7.8. Fractions of 3 mL were collected per test tube at a flow rate of 30 mL/hour at room temperature. All eluted fractions were assessed for their hemorrhagic and phospholipase activities and on SDS-PAGE, as described below. The chromatographic fraction that showed hemorrhagic activity (D4) was selected and lyophilized, and then subjected to ultrafiltration on Vivaspin® 20. The fraction with phospholipase activity (D3) was lyophilized and subjected to a third chromatographic step on a C18 reverse phase column.

#### Ultrafiltration on Vivaspin® 20

A pool of D4 fractions obtained in the chromatographic step on DEAE Sepharose was diluted in 15 mL of Milli-Q water, and desalinated by ultrafiltration on Vivaspin® 20 system, with polyethersulfone membrane with 3,000 MWCO cutoff, by centrifugation at 8,000 × *g* (5804R centrifuge, Eppendorf, Germany) for 20 minutes. The pool was ultrafiltered until the material passing through the iltration membrane showed an optical reading lower than 0.1 Abs at 280 nm, thereby freeing the sample of salts, peptides and other low molecular mass components. Then, the sample had its protein concentration measured by the method of Bradford as described below, and was separated in 1.5 mL conical tubes in volumes equivalent to 1 mg/tube and lyophilized.

#### Reverse phase chromatography (RP-HPLC) on C18 column

Fraction D3 obtained in the chromatographic step on DEAE Sepharose was subjected to a C18 reverse phase column (4.6 mm ID × 25 cm, CLC-ODS, Shimadzu, Japan) using ÄKTA*™* purifier system (GE Healthcare, USA). The column had been previously equilibrated with a solution of 0.1 % trifluoroacetic acid (TFA) (solvent A), and about 10 mg of fraction D3 was diluted in the same solvent and applied to the system using a 500 μL loop. Elution was performed at a flow rate of 0.5 mL/minute with a linear concentration gradient solution containing 70 % acetonitrile and 0.1 % TFA (Solvent B): 0-100 % solvent B in ten column volumes. All eluted fractions were assessed for their phospholipase activity and on SDS-PAGE, as described below. The fraction that showed phospholipase activity was pooled, lyophilized and rechromatographed in the same column, this time using a segmented concentration gradient of 0-60 % solvent B in three column volumes, 60-80 % in five column volumes, and 80-100 % in one column volume.

The purity level of the fraction D4 after Vivaspin® 20 was also evaluated in this reverse phase column using a linear concentration gradient of 0-100 % solvent B in five column volumes.

#### Sodium dodecyl sulfate-polyacrylamide gel electrophoresis (SDS-PAGE)

Chromatographic fractions and isolated toxins were evaluated by SDS-PAGE, performed on 12 % gels using a Mini VE 10 × 10 cm Vertical Gel Electrophoresis System (GE Healthcare, USA), according to Laemmli [16]. Samples were prepared using reducing buffer containing SDS and β-mercaptoethanol, followed by heating at 100 °C for three minutes. After running (15 A, 120 V), the gels were stained with Coomassie brilliant blue R250. The molecular mass standard used was either Spectra Multicolor Broad Range Protein Ladder (10–260 kDa, Thermo Fisher Scientific, Inc., USA) or Unstained Protein Molecular Weight Marker (14.4-116 kDa, Thermo Fisher Scientific, Inc., USA).

#### Protein quantification

Dosages of proteins were performed using Bradford reagent (Sigma-Aldrich, USA), according to the manufacturer instructions, whereas the absorbance of samples was determined at 595 nm in a microplate reader (PowerWave XS2, BioTek, USA). The standard curve was determined from different concentrations (0.1 to 1.5 mg/mL) of bovine serum albumin (BSA).

### Characterization of B. atrox toxins

#### Molecular mass determination

The molecular masses of *B. atrox* toxins were initially estimated according to their SDS-PAGE profile, by interpolating a linear logarithmic curve of the relative molecular mass of standard proteins *versus* the distance of migration of sample proteins in the gel.

MALDI-TOF mass spectrometry analyses were also performed to determine the molecular mass of intact proteins, using an AXIMA Performance MALDI-TOF/TOF mass spectrometer (Shimadzu, Japan) previously calibrated with known molecular mass standards. Mass spectra were acquired in linear mode, evaluating the range from 5,000 to 50,000 m/z. The samples were diluted in 50 μL of Milli-Q water, mixed in a 1:1 ratio with a matrix consisting of sinapinic acid (10 mg/mL) in 50 % acetonitrile and 0.1 % TFA, and applied on the MALDI plate using the dried-droplet method.

#### Isoelectric focusing

The pI of the purified toxins was determined by isoelectric focusing as described by Arantes *et al.* [17]. Briefly, the isoelectric focusing was carried out on a 7 % polyacrylamide gel containing carrier ampholytes (pH 3–10, Sigma-Aldrich). After prefocusing for 30 minutes (settings: 100 V, 30 mA, 5 W), the samples were applied as drops of liquid on the surface of the gel. Standards of isoelectric focusing (IEF Standards, pI range of 4.45 to 9.6, Bio-Rad, USA) were run in parallel to the samples under the same conditions. The isoelectric focusing was performed for approximately four hours (settings: 1500 V, 30 mA, 5 W). Focusing was completed when the voltage reached 1500 V and the current was 2 mA or less. The pH gradient was determined after the current was switched off by cutting sections of the gel (1 × 2 cm) along the gel sides, immersing them individually in 0.5 mL of Milli-Q water for two hours, and measuring their pH. The remaining gel containing the proteins was stained with Coomassie brilliant blue G250. The pI of samples was calculated from the curve of pH versus the distance of migration in the gel.

#### In situ gel digestion and mass spectrometry analysis

MP bands separated by 12 % SDS-PAGE were subjected to *in situ* gel digestion with 0.5 μg of modified trypsin (Promega Co., USA) [18]. The tryptic peptides obtained were desalted in a microtip filled with POROS R2 (Perseptive Biosystems, USA) and eluted with 5 % formic acid in 60 % methanol for analysis. Samples were dried and re-dissolved in 5 μL of α-cyano-4-hydroxycinnamic acid (10 mg/mL), then 2 μL was applied to the MALDI target using the dried-droplet method, followed by analysis by MALDI-TOF MS (AXIMA Performance, Shimadzu Biotech, UK) in the automatic data acquisition mode.

#### N-terminal amino acid sequencing

PLA_2_ sample from RP-HPLC was lyophilized and submitted to Edman degradation [19]. N-terminal amino acid sequencing was performed using a PPSQ-33A automatic sequencer (Shimadzu, Japan). Phenylthiohydantoin (PTH) derivatives of amino acids were identified using an online RP-HPLC by comparison with the retention times of PTH-amino acids of a standard mixture.

#### Amino acid sequence alignment

The amino acid sequences obtained by MALDI-TOF MS and Edman degradation were compared using multiple sequence alignment with other sequences obtained from the NCBI database (http://blast.ncbi.nlm.nih.gov/), using the software ClustalX version 2.0.11. (http://www.clustal.org/).

#### Fibrinolytic activity

The fibrinolytic activity of MP was assessed on fibrin clots formed in Petri dishes, prepared with 0.95 % agarose solution containing 0.3 % fibrinogen and 1 mg/mL thrombin in 50 mM Barbital buffer, pH 7.8, according to Leitão *et al.* [20]. Samples (25 μL) of phosphate-buffered saline (PBS, negative control), *B. atrox* crude venom (20 μg, positive control) or MP (4, 6, 8 and 10 μg) were added to cavities (5 mm diameter) made on the fibrin gel, and incubated at 37 °C for 24 hours. The fibrinolytic activity was evaluated visually and quantified according to the halo diameter (mm).

#### Fibrinogenolytic activity

The ability of MP to digest fibrinogen was evaluated according to the method published by Edgar and Prentice [21], with modifications. Briefly, 25 μL of fibrinogen solution (3 mg/mL in 2 mM Tris–HCl, pH 7.4) was incubated with MP (1 μg) at 37 °C for one hour. The reaction was stopped with 15 μL of 50 mM Tris–HCl, pH 6.8, containing 10 % glycerol (v/v), 4 % SDS (w/v), 0.05 % bromophenol blue (v/v) and 4 % β-mercaptoethanol (v/v), followed by heating at 100 °C for five minutes. After denaturation, one third of the samples (final volume of 75 μL) was assayed by 10 % SDS-PAGE.

#### Hemorrhagic activity

The hemorrhagic activity of chromatographic fractions and the isolated metalloprotease was evaluated by the method described by Nikai *et al.* [22]. Briefly, 50 μL of samples or PBS (negative control) was injected intradermally into the back of BALB/c mice. Inhibition of this activity was evaluated by pre-incubation of enzyme with 5 mM EDTA (ethylenediamine tetraacetic acid) for 30 minutes at 37 °C. After three hours, the animals were euthanized in a CO_2_ chamber, and had their skins removed in order to observe the presence or absence of hemorrhagic halos. The minimum hemorrhagic dose (MHD) was defined as the minimum dose of sample required to induce the formation of a halo with 10 mm diameter.

#### Phospholipase activity

The phospholipase activity of chromatographic fractions and of the isolated phospholipase A_2_ was evaluated on plates, as described by Gutiérrez *et al.* [23], changing the agarose for agar and without using erythrocytes. Briefly, a gel containing 0.01 M CaCl_2_, egg yolk diluted in PBS (pH 7.2) in the ratio 1:3 (v/v), 1 % bacteriological agar and 0.005 % sodium azide was formed in Petri dishes. Then, holes of approximately 5 mm in diameter were made in the gel, and samples were applied at a final volume of 40 μL, followed by incubation at 37 °C overnight. The formation of translucent halos around the holes in the gel was considered to be indicative of phospholipase activity, which was quantified by the measurement of each hole in millimeters. The minimum phospholipase dose (MPD) was defined as the minimum dose of the sample required to induce the formation of a halo with 20 mm diameter.

Modification of residue His48 of the PLA_2_ with 4-bromophenacyl bromide (BPB) was carried out based on previously described methodologies [24]. Briefly, the PLA_2_ (100 μg) was dissolved in 90 μL of 0.1 M ammonium bicarbonate, pH 8.0, and 10 μL of BPB (1 mg/mL in ethanol) was added. The mixture was incubated for 24 hours at 25 °C. After that period, the phospholipase activity of BPB-PLA_2_ (2 μg) was evaluated as described above.

## Results and Discussion

### Purification of *B. atrox* toxins

**Fig. 1:**
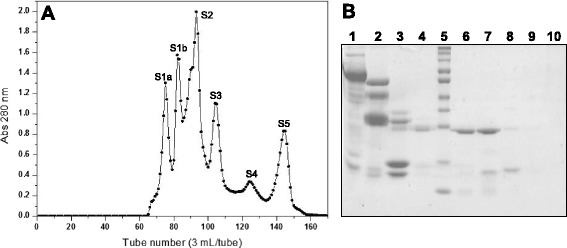
**a** Chromatographic profile of the crude venom of *B. atrox* on a Sephacryl S-200 molecular exclusion column. Elution was performed with 0.2 M ammonium bicarbonate buffer (AMBIC), pH 8.0, collecting 3 mL/tube at a flow rate of 20 mL/hour. **b** 12 % SDS-PAGE. Lanes: 1 – tube 74 (S1a), 2 – tube 82 (S1b), 3 – tube 92 (S2), 4 – tube 99 (S2 valley), 5 – molecular mass standard (260, 140, 100, 70, 50, 40, 35, 25, 15, 10 kDa), 6 – tube 103 (S3), 7 – tube 106 (S3), 8 – tube 115 (S4), 9 – tube 124 (S4), 10 – tube 145 (S5)

The toxins of interest, a P-I class metalloprotease (MP) and an acidic phospholipase A_2_ (PLA_2_), were isolated from *B. atrox* venom by consecutive chromatographic steps, starting the process by performing chromatography on Sephacryl S-200. The chromatographic profile obtained showed six well-defined fractions, identified as S1a, S1b, S2, S3, S4 and S5 (Fig. 1a). Analysis of these fractions by SDS-PAGE showed that fractions S1a, S1b and S2 mainly displayed protein components with molecular masses above 30 kDa. The protein profile of fraction S3 presented bands with molecular masses around 25 kDa and 15 kDa, while fractions S4 and S5 seemed to consist only of components with molecular masses below 15 kDa (Fig. 1b). Fraction S3 was chosen based on its protein profile in SDS-PAGE and the hemorrhagic activity observed, being then subjected to an anion exchange chromatography.

**Fig. 2:**
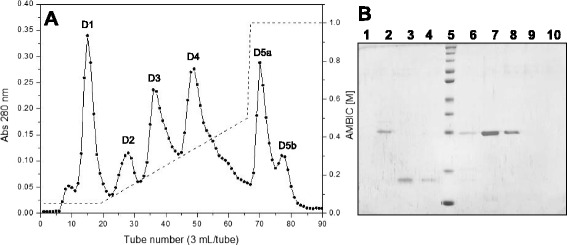
**a** Chromatographic profile of fraction S3 on a DEAE Sepharose anion exchange column. Elution was initiated with 0.05 AMBIC, pH 7.8, followed by a gradient of AMBIC from 0.05 M to 0.5 M, pH 7.8, and finally 1 M AMBIC, pH 7.8. Fractions of 3 mL/tube were collected at a flow rate of 30 mL/hour. **b** 12 % SDS-PAGE. Lanes: 1 – tube 15 (D1), 2 – tube 28 (D2), 3 – tube 36 (D3), 4 – tube 40 (D3), 5 – molecular mass standard (260, 140, 100, 70, 50, 40, 35, 25, 15, 10 kDa), 6 – tube 44 (D3-D4 valley), 7 – tube 49 (D4), 8 – tube 55 (D4), 9 – tube 71 (D5a), 10 – tube 77 (D5b)

DEAE Sepharose chromatography of fraction S3 resulted in six fractions, denominated D1, D2, D3, D4, D5a and D5b (Fig. 2a). Several of these fractions (D1, D5a and D5b) showed no visible bands on SDS-PAGE, indicating the presence of only low-molecular-mass components. Fraction D2 showed a protein band with molecular mass slightly above 25 kDa, whereas fractions D3 and D4 showed single bands around 15 kDa and 25 kDa, respectively (Fig. 2b). Based on these protein profiles and the hemorrhagic and phospholipase activities, it was determined that the PLA_2_ was in fraction D3 and the MP in fraction D4. Thereafter, two separate isolation procedures were used to obtain each enzyme.

**Fig. 3:**
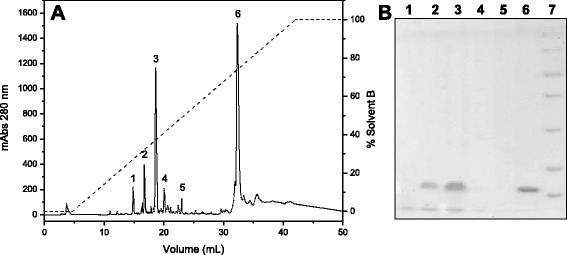
**a** Chromatographic profile of fraction D3 on a C18 reverse phase column. Elution was performed using a RP-HPLC system at a flow rate of 0.5 mL/minute with a linear concentration gradient of 0-100 % solvent B (70 % acetonitrile and 0.1 % TFA) in ten column volumes. **b** 12 % SDS-PAGE. Lanes: 1 – fraction 1; 2 – fraction 2; 3 – fraction 3; 4 – fraction 4; 5 – fraction 5; 6 – fraction 6; 7 – molecular mass standard (116, 66.4, 45, 35, 25, 18, 4, 14.4 kDa)

**Fig. 4:**
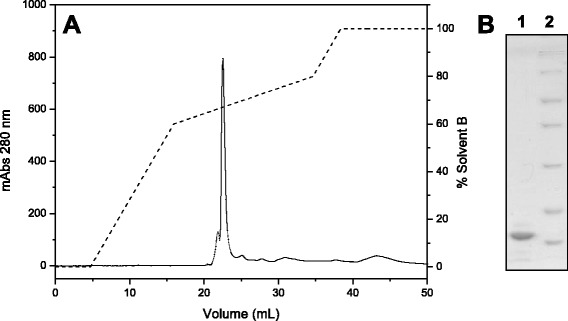
**a** Chromatographic profile of fraction 6 on a C18 reverse phase column. Elution was performed using a RP-HPLC system at a flow rate of 0.5 mL/minute using a segmented concentration gradient of 0-60 % solvent B in three column volumes, 60-80 % in five column volumes and 80-100 % in one column volume. **b** 12 % SDS-PAGE. Lanes: 1 – PLA_2_, 2 – molecular mass standard (116, 66.4, 45, 35, 25, 18.4, 14.4 kDa)

Fraction D3, which contained the PLA_2_, was applied to a C18 reverse phase column, resulting in six major fractions (Fig. 3a). This chromatographic step enabled the separation of the acidic and catalytically active PLA_2_ from other fractions containing low molecular mass peptides (fractions 1, 4 and 5) which do not appear on 12 % SDS-PAGE, and from phospholipases A_2_ without catalytic activity (fractions 2 and 3) (Fig. 3b) The fraction that showed phospholipase activity (fraction 6), indicative of catalytic activity related to the residue Asp49, was then rechromatographed on the same column to assess its purity level. The chromatographic profile shows a fraction eluted around 65 % solvent B (Fig. 4a), which appeared as a single band of approximately 14 kDa on SDS-PAGE (Fig. 4b).

**Fig. 5:**
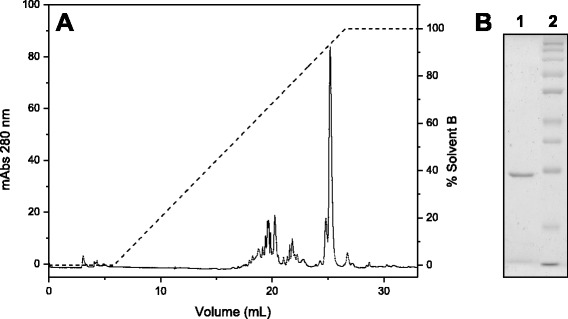
**a** Evaluation of the purity level of fraction D4 after Vivaspin® 20 on a C18 reverse phase column. After having been subjected to ultrafiltration on a Vivaspin® 20 system, fraction D4 was evaluated by RP-HPLC using a C18 column with a linear concentration gradient of 0-100 % solvent B in five column volumes. **b** 12 % SDS-PAGE. Lanes: 1 – MP, 2 – molecular mass standard (260, 140, 100, 70, 50, 40, 35, 25, 15, 10 kDa)

Fraction D4 containing the MP was ultrafiltered on a Vivaspin® 20 system, which was used as a third step of isolation and may be considered a molecular exclusion step, since it enabled clearance of the fraction of low-molecular-mass compounds (such as peptides) and salts from the anion exchange chromatographic step. After this ultrafiltration step, the MP purity level was evidenced by RP-HPLC using a C18 column, eluting with ~90 % solvent B (Fig. 5a), and appearing as a single band of molecular mass around 25 kDa on SDS-PAGE (Fig. 5b). Usually, reverse phase chromatographic steps are used only to verify the purity levels of metalloproteases, since these enzymes lose their proteolytic activity when exposed to organic solvents such as TFA and acetonitrile, possibly due to denaturation of the molecules promoted by the low pH of solvents.

Purification of P-I class metalloproteases is commonly performed using two to three chromatographic steps, with a predominance of molecular exclusion and ion exchange steps. A purification process employing a single chromatographic step was described for neuwiedase from *B. neuwiedi* venom, nevertheless, most procedures usually comprise two steps, as described for BaP1 from *B. asper*, leucurolysin-a from *B. leucurus*, atroxlysin-I from *B. atrox* and BjussuMP-II from *B. jararacussu* venom [25–29]. Some studies also show the isolation of P-I metalloproteases using three chromatographic steps, as described for BmooMP-α from *B. moojeni* and for BpirMP from *B. pirajai* venom, which were isolated by combining molecular exclusion, ion exchange and affinity steps [30, 31].

Although our research group had already proposed a method for the isolation of a P-I metalloprotease denominated Batroxase from *B. atrox* venom using a molecular exclusion step on Sephadex G-75 and an anion exchange chromatography on ES-502 N 7C column [32], the new method described in the present study was standardized so that an acidic phospholipase A_2_ could also be obtained from this venom. By using this novel methodology, a P-I metalloprotease and an acidic PLA_2_ were successfully isolated from *B. atrox* venom using the same two initial chromatographic steps and a third distinct one for each enzyme.

Acidic PLA_2_s from *Bothrops* venoms are commonly purified by a combination of chromatographic methods, including molecular exclusion, ion exchange, RP-HPLC and hydrophobic steps. Cogo *et al.* [33] isolated two acidic PLA_2_s from *B. insularis* venom using a single RP-HPLC step. Other enzymes were isolated using two chromatographic steps, as described for BthA-I-PLA_2_ from *B. jararacussu*, BpirPLA_2_-I from *B. pirajai*, BL-PLA_2_ from *B. leucurus*, BmooPLA_2_ from *B. moojeni* and BaSPIIRP4 from *B. alternatus* venom [24, 34–37]. There are also reports of the combination of three or four chromatographic steps for obtaining some acidic PLA_2_s from *Bothrops* venoms [38–40].

After the isolation of *B. atrox* toxins, some biochemical and functional experiments were performed in order to identify the enzymes of interest, including the determination of molecular masses, isoelectric points, partial amino acid sequences and evaluation of characteristic functional activities for metalloproteases and phospholipases A_2_.

### Characterization of the MP

MP showed molecular mass of 22.9 kDa by MALDI-TOF MS and 26.2 kDa when estimated by SDS-PAGE. P-I SVMPs present variable molecular masses ranging from 20 to approximately 30 kDa, e.g. neuwiedase (20 kDa), BthMP (23 kDa), leucurolysin-a (23 kDa), atroxlysin-I (23 kDa), BpirMP (23 kDa), BaP1 (24 kDa), BnP1 (24 kDa), BH2 (26 kDa), Batroxase (27 kDa by SDS-PAGE and 22.9 kDa by MALDI-TOF MS) and BaltMP-I (29 kDa) [25–28, 31, 32, 41–44]. Nevertheless, it should be taken into account that some of those differences in the molecular masses could be attributable to the different sensitivity of the methodologies used for their resolution, e.g. SDS-PAGE or MALDI-TOF MS.

**Fig. 6:**
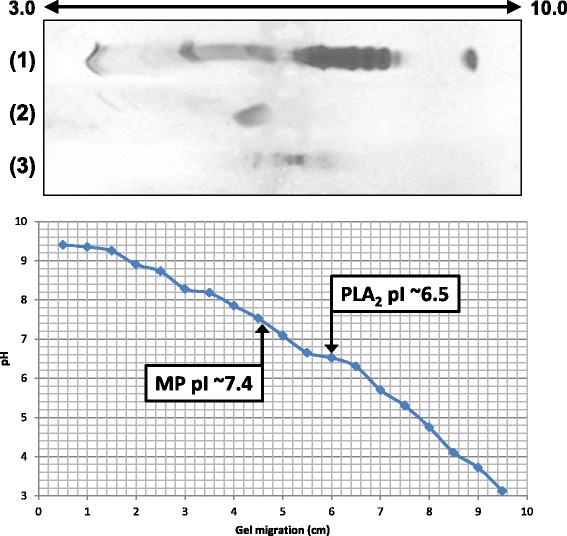
Isoelectric focusing of the toxins isolated from *B. atrox* venom on a 7 % polyacrylamide gel. Samples: (1) isoelectric focusing standard (IEF Standards, pI ranges from 4.45 to 9.6, Bio-Rad, USA), (2) PLA_2_ (pI ~6.5) and (3) MP (pI ~7.4). The pI of samples was calculated from the curve of pH versus the migration distance in the gel

The isoelectric focusing showed that MP is a neutral protein with pI of approximately 7.4 (Fig. 6). P-I metalloproteases may present pI values ranging between 5 and 8, and thus may show acidic character such as neuwiedase, BjussuMP-II and BH2, neutral character as Batroxase and BthMP or basic character as BJ-PI2 [25, 29, 32, 41, 43, 45].

**Fig. 7:**
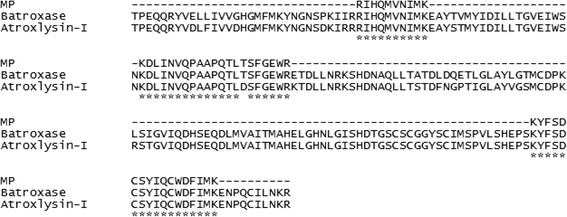
Comparison of the partial amino acid sequence obtained for the MP from *B. atrox* venom with two other P-I metalloproteases previously isolated from the same venom: Batroxase and atroxlysin-I. Multiple sequence alignment was made using the program ClustalX v. 2.0.11. (*) indicates positions with fully conserved amino acid residues

Partial amino acid sequencing revealed high similarity between MP and other P-I metalloproteases previously isolated from *B. atrox* venom, such as atroxlysin-I and Batroxase, with 100 % identity with the latter enzyme (Fig. 7) [28, 32]. SVMPs are classified according to their structural domains: P-I class, presenting only the metalloprotease domain; P-II class, presenting the metalloprotease domain and the disintegrin domain; P-III class, containing the disintegrin domain, cysteine rich domain and the metalloprotease domain [46]. Thus, P-I SVMPs belong to the simplest class of metalloproteases, with lower molecular masses and an average of 200–210 amino acid residues [47]. The zinc binding catalytic site of these molecules is formed by the consensus sequence HEXXHXXGXXH, with the conserved Met-turn sequence CI/VM adjacent to the site [48, 49]. Although these portions were not determined in the partial sequencing of the MP from *B. atrox* venom, the multiple alignment (Fig. 7) and the biochemical and functional characteristics confirm that it is a P-I class metalloprotease.

**Fig. 8:**
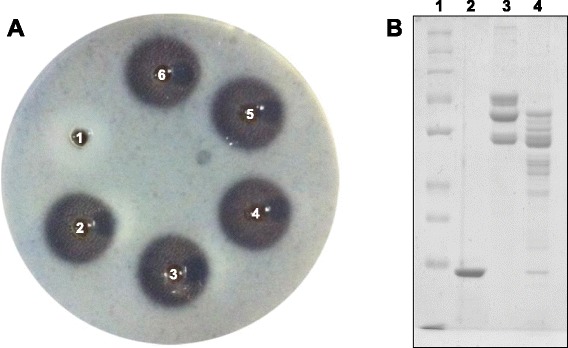
Fibrin(ogen)olytic activity of the MP isolated from *B. atrox* venom. **a** Evaluation of the fibrinolytic activity on fibrin gel. Samples were applied on plates containing fibrin gels, and incubation was performed at 37 °C for 24 h. Samples (halo diameter): 1. PBS (0 mm), 2. *B. atrox* venom 20 μg (15 mm), 3. MP 4 μg (15 mm), 4. MP 6 μg (15.5 mm), 5. MP 8 μg (16 mm), 6. MP 10 μg (16.5 mm). **b** Evaluation of the fibrinogenolytic activity by SDS-PAGE. Samples were incubated at 37 °C for 1 h and then evaluated on 10 % polyacrylamide gel under denaturing conditions. Lanes: 1 Molecular mass standard (260, 140, 100, 70, 50, 40, 35, 25, 15 kDa), 2. MP 5 μg, 3. Fibrinogen 25 μg, 4. Fibrinogen 25 μg + MP 1 μg

In relation to the functional characterization, MP showed high fibrin(ogen)olytic activity, with low doses inducing significant fibrinolysis halos (Fig. 8a) and preferential degradation of the Aα chains of fibrinogen, although it also induced degradation of the Bβ chains (Fig. 8b).

Most P-I SVMPs are fibrinogenolytic enzymes that preferentially degrade the Aα chains of fibrinogen, while also degrading the Bβ chains at slower ratios [6]. Examples of fibrinogenolytic metalloproteases from *Bothrops* venoms include BthMP, BmooMP-α, atroxlysin-I, Batroxase, BaltMP-I, BaP1, neuwiedase and BpirMP [25, 26, 28, 30–32, 41, 44]. Some SVMPs also showed fibrinolytic activity, including BnP1, bothrojaractivase, BthMP, BpirMP, atroxlysin-I and Batroxase [28, 31, 32, 41, 42, 50].

Recent studies have been exploring the potential of fibrin(ogen)olytic SVMPs as thrombolytic agents, since these enzymes may act directly on fibrin clots, and also promote depletion of fibrinogen molecules and thus prevent the formation of new clots. For this reason, fibrin(ogen)olytic SVMPs with low or no hemorrhagic activity, such as fibrolase and its recombinant form alfimeprase, have been evaluated as potential drugs for the treatment of patients with vascular diseases [51].

**Fig. 9:**
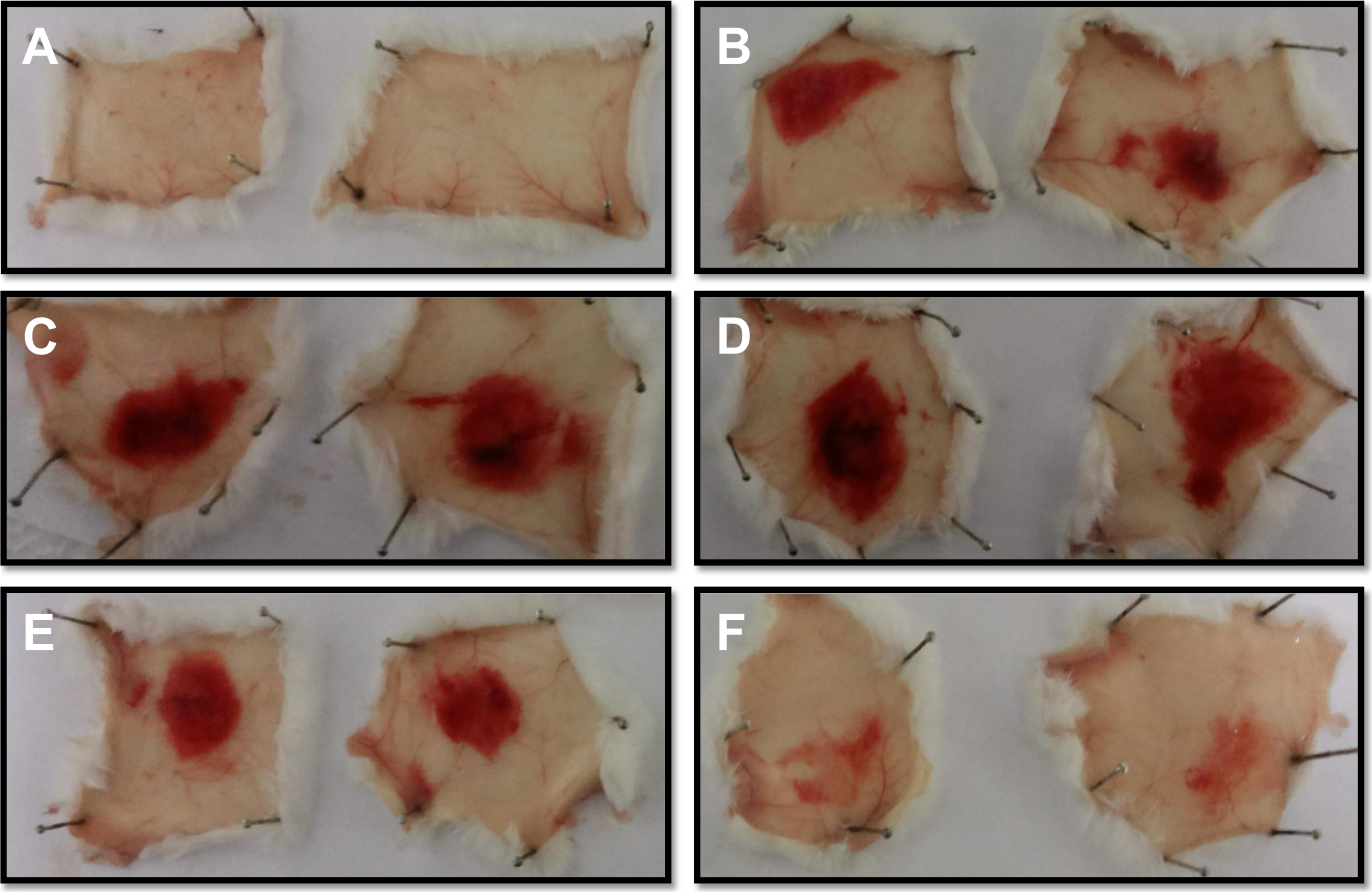
Evaluation of the hemorrhagic activity of the MP from *B. atrox* venom. Samples (n = 2) were injected intradermally into the back of BALB/c mice, and after three hours, the animals were euthanized and had their skins removed to allow the observation of the presence or absence of hemorrhagic halos. Samples (halo diameter): (**a**) PBS (0 mm), (**b**) MP 5 μg (10 mm, determined as the MHD), (**c**) MP 10 μg (16 mm), (**d**) MP 20 μg (24 mm), (**e**) MP 5 μg + PLA_2_ 1:1 (12 mm), (**f**) MP 5 μg + 5 mM EDTA (0 mm)

The MP from *B. atrox* venom showed high hemorrhagic activity at doses up to 20 μg, with MHD close to 5 μg and significant inhibition observed after incubation with 5 mM EDTA (Fig. 9). This inhibition promoted by chelating agents such as EDTA and 1,10-phenanthroline on the proteolytic and hemorrhagic activities of SVMPs is associated with the chelation of the zinc ion, which is essential for the catalytic activity of these molecules [26, 43].

The hemorrhage induced by SVMPs is related to the hydrolysis of basal membrane of capillaries [52]. In relation to hemorrhagic potential, P-III SVMPs are the most potent among the three classes, being capable of inducing not only local but also systemic hemorrhages, while P-I SVMPs induce mostly local hemorrhaging [53]. Additionally, differences in the hemorrhagic potential of P-I SVMPs can also be observed [54, 55], with some enzymes presenting this activity [26, 28, 32, 41, 56] while others do not [25, 29, 30]. The comparison between structures of hemorrhagic and non-hemorrhagic P-I SVMPs showed small variations in loop regions around the catalytic site, suggesting that the variations of hemorrhagic potential may be related to such areas [57].

Some P-I metalloproteases have already been described from *B. atrox* venom, e.g. HI-5, atroxlysin-I, BaTx-I and Batroxase [28, 32, 56, 58]. As not all of these enzymes had their amino acid sequences elucidated, it is possible that some of them are the same molecule described by different authors. It is also possible that the different geographical locations of *B. atrox* specimens used as venom sources in these different studies (i.e. Brazil, Peru and Colombia) exerted some type of influence on the structural and functional differences reported for these enzymes, as shown by proteome studies on venoms of this snake species [14].

Considering the characteristics found for the MP from *B. atrox* described in the present study, it was noted that this enzyme showed significant similarities to Batroxase, including partial amino acid sequence, molecular mass (determined by both SDS-PAGE and MALDI-TOF MS methods), neutral pI and fibrin(ogen)olytic and hemorrhagic activities [32]. Thus, our findings suggest that the isolated MP can be identified as Batroxase. 

### Characterization of the PLA_2_

**Fig. 10:**
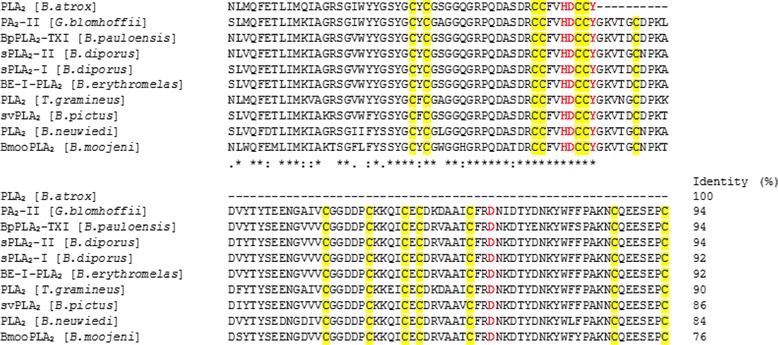
Multiple alignment of the N-terminal sequence of the PLA_2_ from *B. atrox* venom with other acidic PLA_2_s from snake venoms. The highlighted amino acid residues are part of disulfide bonds (yellow) and the catalytic site (red). GI numbers (from top to bottom, respectively): 129420, 528050010, 387537880, 387537878, 123907441, 3914270, 23396779, 458601843 and 403399514. (*) indicates positions with fully conserved amino acid residues; (:) indicates conservation of the following groups with high score: K/R/Q/H, S/A, K/N/D, F/L/V/I, E/D/N/Q, T/S/A, I/M/L; (.) indicates conservation of the following groups with lowest score: N/R/G, G/D/N, A/V/T, Q/K/E/R, S/K/G/A, D/K/H, C/S, T/P

*B. atrox* PLA_2_ presented a molecular mass of 13.7 kDa by MALDI-TOF MS and 14.4 kDa by SDS-PAGE, and was revealed to be an acidic enzyme with pI ~ 6.5 (Fig. 6). Acidic snake venom PLA_2_s present molecular masses around 14 kDa and pI varying from 4.0 to 5.5. Some examples are BthA-I-PLA_2_ with 13.7 kDa and pI 4.5, BpirPLA_2_-I with 13.7 kDa and pI 4.8, Bp-PLA_2_ with 15.8 kDa and pI 4.3, BmooPLA_2_ with 13.6 kDa and pI 5.2 and BL-PLA_2_ with 15 kDa and pI 5.4 [24, 34–36, 59]. None of these acidic PLA_2_ showed a pI value superior to 5.5 as shown for the PLA_2_ isolated in this study (pI ~ 6.5). Although this pI value is close to neutral, alignment with other PLA_2_s confirmed its high similarity to acidic proteins, including PA2-II from *Gloydius blomhoffii* venom (gi 129420) and BpPLA_2_-TX-I from *B. pauloensis* venom [40] (Fig. 10).

PLA_2_s are classified into 15 different subgroups according to specific features, with snake venom PLA_2_s belonging to groups I and II [60, 61]. Group IIA PLA_2_s are further divided into Asp49 PLA_2_s, enzymes that usually exhibit high catalytic activity, and Lys49 PLA_2_s with low or no enzymatic activity on artificial substrates [62, 63]. The catalytic site of PLA_2_s is composed of the residues His48, Asp49, Tyr52 and Asp99, and the majority of these enzymes have conserved His48 and Asp49 residues that present a catalytically essential water molecule attached to their side-chains by hydrogen bonds [64, 65]. Besides the catalytic site, the calcium binding site (X27CGXGG32) is also present in all group II catalytically active PLA_2_s [66]. Three active site residues (His48, Asp49 and Tyr52), the calcium binding site and six conserved cysteine residues related to the formation of disulfide bonds were determined in the partial amino acid sequencing of *B. atrox* PLA_2_ (Fig. 10).

The PLA_2_ showed high phospholipase activity at doses up to 8 μg, with MPD of 2 μg (Table 1). These results show a high catalytic activity for this enzyme, which is consistent with other acidic PLA_2_s from *Bothrops* venoms, such as BmooTX-I, with MPD of 1 μg, and BpirPLA_2_-I, with MPD of 4 μg [34, 38]. Some studies describe the acidic Asp49 PLA_2_s as catalytically more active than the basic isoforms [24, 34, 38, 39, 59]. Additionally, treatment with BPB significantly inhibited the phospholipase activity of the PLA_2_ from *B. atrox* (Table 1), as already described for other acidic PLA_2_s [34, 38].

**Table 1:** Phospholipase activity of *B. atrox* venom and the isolated PLA_2_

Samples	Halo diameter (mm) (mean ± SEM) (n = 3)
PBS	0
*B. atrox* (5 μg)	17.5 ± 0.5
*B. atrox* (10 μg)	20.0 ± 0.5
PLA_2_ (0.5 μg)	14.5 ± 0.5
PLA_2_ (1 μg)	16.5 ± 0.5
PLA_2_ (2 μg) *	20.0
PLA_2_ (4 μg)	21.0 ± 0.5
PLA_2_ (8 μg)	22.0
PLA_2_ (2 μg) + BPB	6.5 ± 0.5
PLA_2_ (2 μg) + MP (1:1)	20.5 ± 0.5

* Determined as the minimum phospholipase dose (MPD)

Some phospholipase A_2_ enzymes were previously described from *B. atrox* venom, e.g. a myotoxin with phospholipase and anticoagulant activity, basic and neutral Asp49 PLA_2_s, named BaPLA_2_I and BaPLA_2_III and a Lys49 PLA_2_ called myotoxin I [67–69]. Recently, a study published by Furtado *et al.* [70] described the isolation of three PLA_2_s from *B. atrox* venom: a Lys49 (BaTX-I), an Asp49 (BaTX-II) and an acidic Asp49 myotoxin (BaPLA_2_). However, as the authors did not perform amino acid sequencing of the molecules, it is not possible to determine whether these enzymes are novel or the same ones previously described in the literature. Thus, as there are no reports in the literature of acidic PLA_2_s isolated from *B. atrox* venom, we denominated the enzyme described in the present study BatroxPLA_2_, an unprecedented acidic phospholipase A_2_.

### Synergism

It is well known that snake toxins can act independently or synergistically [8]. To assess this possible synergism between the MP and the PLA_2_ from *B. atrox*, the hemorrhagic and phospholipase activities were evaluated using a mixture of the enzymes at the molar ratio of 1:1, which resulted in no significant changes compared to the isolated activities of each enzyme (Fig. 9 and Table 1), suggesting that those activities were not enhanced or impaired by the combination of both toxins. Future studies will further evaluate the possible synergistic effects of the two toxins on different physiological processes, e.g. inflammation.

## Conclusions

The interest in the biochemical and functional characterization of toxins isolated from snake venoms is due not only to their relevance in envenomations, but also to their potential use as valuable research tools in different areas of knowledge. Pharmacological and biochemical studies conducted in recent decades have shown the presence of a variety of enzymes, toxins and biologically active compounds in these venoms, as well as the great diversity of their actions. Consequently, numerous attempts have been made to use these compounds as tools for research and for applications in the medical field, and as such, the purification and characterization of snake toxins are of utmost importance.

In this context, the present study successfully standardized a purification procedure, mainly composed of classical chromatographic techniques, for the isolation of a P-I metalloprotease identified as Batroxase and a new acidic PLA_2_ denominated BatroxPLA_2_ from *B. atrox* venom. These two enzymes will be used in future studies to evaluate their effects on the complement system and the inflammatory process, in addition to the thrombolytic potential of the metalloprotease.

### Ethics committee approval

Animal care procedures were performed according to the Brazilian College of Animal Experimentation (COBEA) guidelines and the experimental protocols were approved by the Committee for Ethics on Animal Use (CEUA) from FCFRP-USP (protocol number: 13.1.336.53.4).
